# Balancing Bleeding and Clot: Type 2 Heparin-Induced Thrombocytopenia With Extensive Thromboses in a Patient With Subdural Hematoma

**DOI:** 10.7759/cureus.99999

**Published:** 2025-12-24

**Authors:** George K Annan, Enoch Enninful, Chinenye Egwuonwu, Stanley E Atencah, Patrick O Berchie

**Affiliations:** 1 Internal Medicine, Piedmont Athens Regional Medical Center, Athens, USA; 2 Plastic and Reconstructive Surgery, Korle Bu Teaching Hospital, Accra, GHA

**Keywords:** argatroban, atrial fibrillation, deep vein thrombosis (dvt), direct oral anticoagulants (doac), dvt, essential thrombocythemia, heparin-induced thrombocytopenia (hit), noac therapy, pulmonary embolism (pe), subdural hematomas

## Abstract

Heparin-induced thrombocytopenia (HIT) type 2 is a rare, immune-mediated complication of heparin therapy characterized by thrombocytopenia and paradoxical thrombosis. It results from platelet-activating antibodies against platelet factor 4 (PF4)/heparin complexes and can precipitate life-threatening thromboembolic events. Management becomes especially challenging in patients with concurrent bleeding, such as intracranial hemorrhage, where anticoagulation carries significant risk.

An 87-year-old man with essential thrombocythemia and atrial fibrillation on rivaroxaban presented with a right subacute subdural hematoma after a fall. Anticoagulation was held, and he underwent burr hole evacuation with middle meningeal artery embolization, receiving prophylactic heparin postoperatively. Three weeks later, he developed a left leg deep vein thrombosis (DVT); therapeutic heparin was initiated but stopped after >50% platelet decline and hematoma progression. With a high-probability 4Ts score, an inferior vena cava (IVC) filter was placed, and he was discharged from anticoagulation pending HIT results. Six days later, he re-presented with dyspnea and limb swelling. Imaging revealed bilateral pulmonary embolism and extensive DVTs in all extremities. HIT was confirmed by positive PF4 enzyme-linked immunosorbent assay (ELISA) and serotonin release assay. He was started on argatroban and later transitioned to apixaban 5 mg twice daily once hematoma stability was confirmed. He remained neurologically stable on follow-up.

This case underscores the therapeutic dilemma of managing HIT in the setting of active intracranial hemorrhage. While immediate anticoagulation is required to mitigate thrombosis, it risks worsening intracranial bleeding. Argatroban, due to its short half-life, provides a safe bridge in such scenarios. Transitioning to apixaban after radiographic stability reflects a growing trend toward individualized use of direct oral anticoagulants (DOACs) in the management of HIT. Notably, the coexistence of essential thrombocythemia and atrial fibrillation may have further amplified both thrombotic and bleeding risk, emphasizing the possible limitations of existing scoring tools such as the 4Ts in complex hematologic settings.

In patients with type 2 HIT and active intracranial bleeding, anticoagulation may still be necessary to prevent fatal thromboses. Tailored multidisciplinary management can achieve favorable outcomes. This case highlights the need for further research into risk stratification frameworks that integrate concurrent hematologic disorders and competing thrombotic-hemorrhagic conditions to guide safe anticoagulant selection and timing in type 2 HIT, especially with a concurrent bleed.

## Introduction

Type 2 heparin-induced thrombocytopenia (HIT) is an immune-mediated, prothrombotic disorder driven by IgG antibodies against platelet factor 4 (PF4)/heparin complexes, leading to thrombocytopenia and platelet activation via FcγRIIa receptors, resulting in a hypercoagulable state. It typically develops five to 10 days after heparin exposure. This paradoxical prothrombotic response can lead to venous and arterial thromboses despite low platelet counts [1-3].

The incidence generally ranges from 0.1% to 5% in surgical patients and about 0.2% to 3% in all patients with heparin exposure. Patients receiving unfractionated heparin (UFH) have the highest risk, with incidence rates of 1-5% reported [1,4-7]. Thrombocytopenia is the hallmark. Thromboembolic complications occur in up to 50% of patients, with venous thromboembolism being more common than arterial thrombosis [4].

Diagnosis begins with the application of pretest clinical tools. The 4Ts scoring system is the most widely used tool to assess the likelihood of HIT. High and intermediate scores prompt urgent discontinuation of heparin, consideration of alternative non-heparin anticoagulation, and further laboratory testing [8,9]. Immunoassays for PF4/heparin antibodies are highly sensitive and used as screening tests for HIT. Confirmation requires functional assays, such as the serotonin release assay (SRA) or the heparin-induced platelet aggregation assay, because of their high specificities [4,10].

Immediate discontinuation of all heparin products and initiation of anticoagulation with a non-heparin agent, irrespective of the presence of thrombosis, remains the cornerstone of HIT management [3,9]. Preferred non-heparin anticoagulants include argatroban, bivalirudin, danaparoid, fondaparinux, or a direct oral anticoagulant (DOAC), with the choice determined by drug availability, patient comorbidities (renal or hepatic dysfunction), bleeding risk, and clinical stability. In critically ill patients or those at high risk for bleeding or urgent procedures, argatroban or bivalirudin is preferred. Due to their short half-lives, they allow rapid titration and cessation if bleeding occurs [9,11,12]. Transition to DOACs such as apixaban or rivaroxaban is increasingly recognized as a safe long-term strategy once bleeding stability is achieved [9,11].

While standard management involves discontinuation of heparin and initiation of non-heparin anticoagulants, clinical decision-making becomes particularly challenging in patients with active bleeding, such as intracranial hemorrhage, where the risk-benefit balance is complex. This challenge is further amplified in patients with comorbid conditions like essential thrombocythemia and atrial fibrillation, which independently increase both thrombotic and hemorrhagic risk. This case report describes an elderly man with essential thrombocythemia and atrial fibrillation who developed widespread thromboses due to type 2 HIT in the setting of an acute-on-chronic subdural hematoma. The case highlights the delicate balance between thrombosis management and hemorrhage control and emphasizes the need for individualized, multidisciplinary care. It also highlights the absence of integrated scoring tools in complex hematologic settings and comorbid conditions with heightened thrombotic and bleeding risks, as seen in our patient with essential thrombocythemia and atrial fibrillation.

## Case presentation

An 87-year-old man with essential thrombocythemia and atrial fibrillation on rivaroxaban presented after a fall with progressive headache and confusion. Head CT revealed a right subdural hematoma with midline shift. Rivaroxaban was held. Neurosurgery performed burr-hole evacuation due to mass effect and neurologic symptoms, and middle meningeal artery embolization was added to reduce the high risk of hematoma recurrence given the patient’s age, essential thrombocythemia, and anticipated need for future anticoagulation. Postoperatively, he received prophylactic-dose subcutaneous UFH for approximately seven days. A routine postoperative head CT demonstrated expected postsurgical changes with decreased hematoma volume and no new bleeding or mass effect. Clinically, the patient’s headache and confusion improved, and he returned to his neurological baseline, allowing for discharge home.

Three weeks after discharge, he presented with left lower extremity swelling. Doppler ultrasound revealed an acute deep vein thrombosis (DVT) in the left lower extremity. A continuous heparin infusion was initiated but discontinued after a greater than 50% platelet decline and subdural hematoma progression, in contrast to the stable postoperative study. The abrupt drop in platelets from 131×10⁹/L to 44×10⁹/L within 24 hours raised immediate concern for HIT. This platelet trend, combined with the patient’s new thrombosis, timing of exposure, and high probability of HIT on 4Ts scoring, prompted cessation of all heparin products and urgent laboratory testing. Given the recent subdural hematoma, an extensive risk-versus-benefit discussion on initiation of a non-heparin anticoagulant was held with the patient, who elected to defer treatment pending the results of HIT testing. An inferior vena cava (IVC) filter was placed, and he was discharged from anticoagulation with close hematology follow-up pending results of HIT testing. 

**Figure 1:**
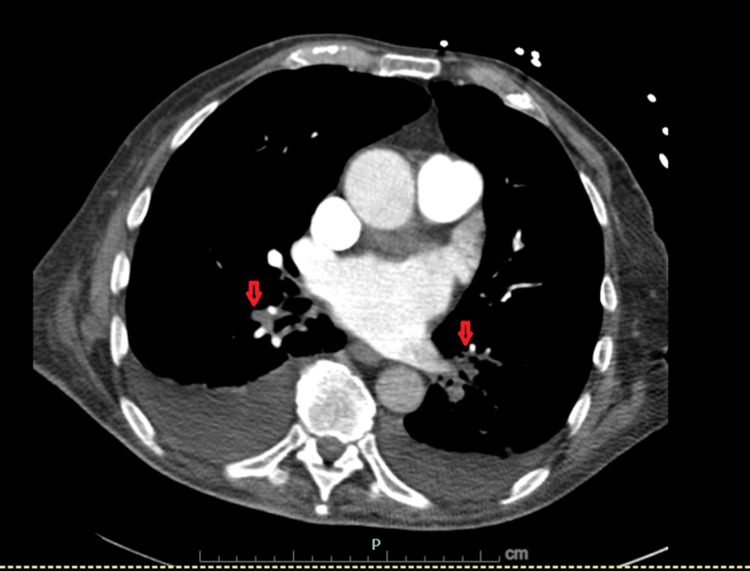
CTPA demonstrating bilateral segmental pulmonary emboli CTPA showing filling defects (red arrows) within the left lower lobe medial basal and right lower lobe lateral basal segmental branches, consistent with bilateral pulmonary emboli. Pulmonary artery caliber is normal. CTPA, CT pulmonary angiogram

**Figure 2:**
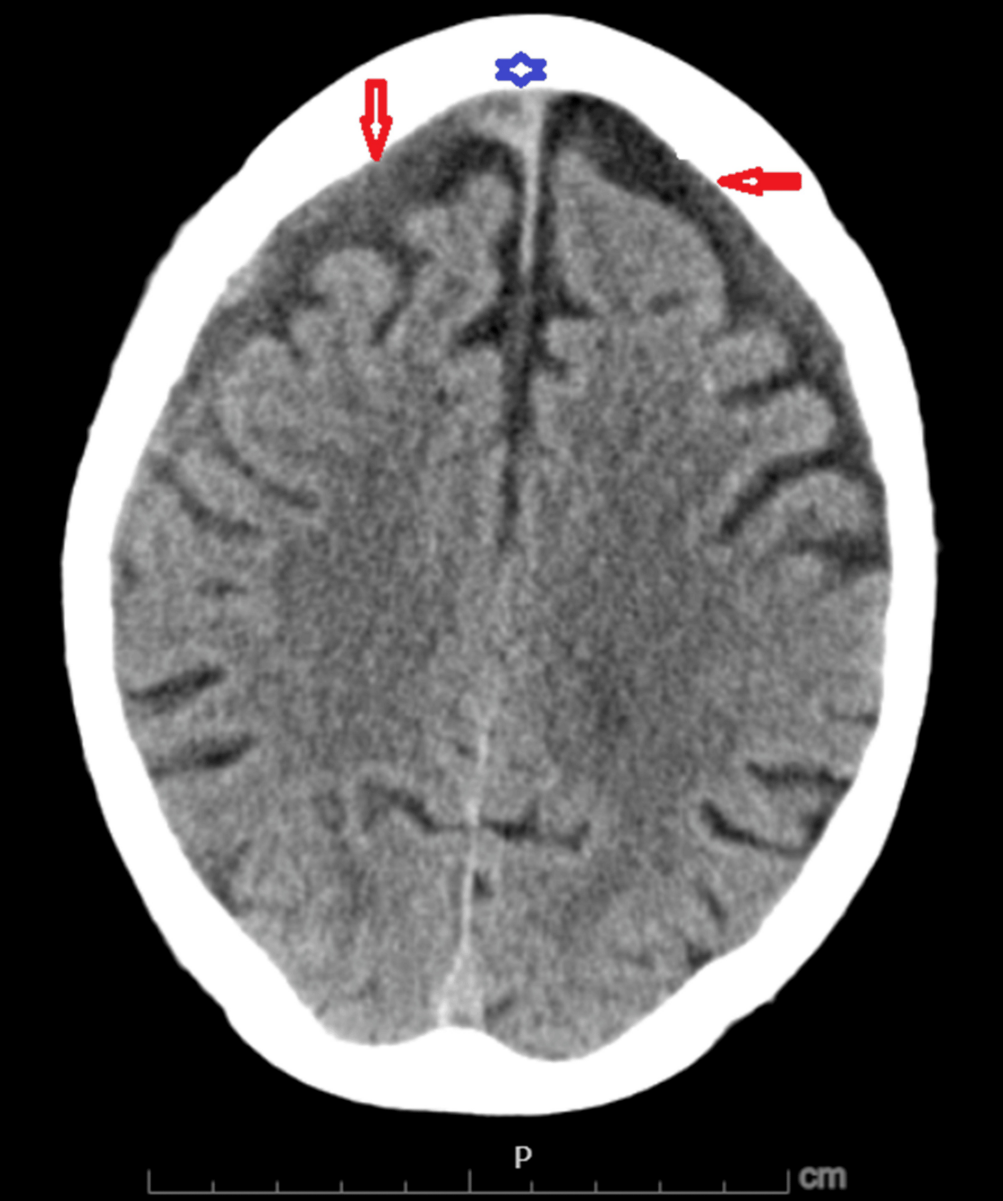
Non-contrast head CT showing acute-on-chronic bilateral subdural hematomas Axial non-contrast CT of the head demonstrating bilateral subdural hematomas. Chronic components are indicated by red arrows, while acute hyperdense components on the right are marked with a blue star. The maximal thickness measures 11 mm on the right and seven mm on the left. There is no significant midline shift or hydrocephalus.

Six days later, he re-presented with dyspnea and swelling of all extremities. CT pulmonary angiography (CTPA) showed bilateral pulmonary emboli (Figure 1), and Doppler ultrasound confirmed extensive DVTs in all four limbs. Head CT scan demonstrated chronic bilateral subdural hematomas with a small acute right-sided component (Figure 2). Laboratory studies showed thrombocytopenia with a platelet count of 80×10⁹/L. HIT antibody testing was strongly positive by PF4 enzyme-linked immunosorbent assay (ELISA) (optical density 2.449), and a confirmatory SRA revealed >60% serotonin release at low-dose heparin, suppressed at high-dose, confirming type 2 HIT. The concordant PF4 and SRA results confirmed HIT, establishing that the ongoing thrombosis was immunologically driven rather than incidental or due to immobility alone. A summary of the pertinent laboratory trends, including platelet count and confirmatory HIT testing, is provided in Table 1.

**Table 1:** Key laboratory findings during hospital course Values represent temporal changes in platelet count and hemoglobin during the patient’s hospitalization and follow-up. PF4 ELISA and serotonin release assay confirmed the diagnosis of type 2 HIT during week 4. PF4, platelet factor 4; ELISA, enzyme-linked immunosorbent assay; HIT, heparin-induced thrombocytopenia

Parameter	Day 0	Week 3	Week 4	Week 6 (follow up)	Reference ranges
Platelet count, ×10⁹/L	160	131>44	80	154	150-400
Hemoglobin, g/dL	12.2	11.0	10.2	10.6	13.0-17.0
PF4 ELISA (optical density)			2.449		<0.4
Serotonin release assay			>60%, suppressed at high dose		<20%

After multidisciplinary discussion among hematology, neurosurgery, and internal medicine, argatroban infusion was initiated with close neurological monitoring. Serial head CTs demonstrated no increase in hematoma thickness, no new hyperdense components, and unchanged midline shift measurements, indicating radiographic stability. The patient was transitioned to apixaban 5 mg twice daily without a loading dose. He was discharged home on oral anticoagulation with close follow-up. He remained neurologically stable on follow-up.

## Discussion

HIT is an immune-mediated condition in which platelet activation leads to both thrombocytopenia and paradoxical thrombosis [1-3]. The condition can lead to devastating complications, including DVT, PE, stroke, limb gangrene, and adrenal hemorrhage [4]. Our patient’s presentation was further complicated by essential thrombocythemia, a chronic myeloproliferative neoplasm associated with increased thrombotic and bleeding risk, and by concurrent atrial fibrillation requiring anticoagulation. Notably, patients with essential thrombocythemia, particularly those carrying the JAK2 V617F mutation, may have an increased risk of HIT-associated thrombosis, highlighting the potential synergistic effect of essential thrombocythemia and HIT in promoting extensive thrombotic complications [13]. Currently, there are no specific integrated risk tools for patients with these comorbid conditions diagnosed with HIT.

The diagnosis of HIT relies on a combination of clinical probability assessment using the 4Ts score and confirmatory laboratory testing [8,9]. Our patient had a 4Ts score of 8, signifying a high probability of HIT and necessitating laboratory testing. Immunoassays such as PF4 ELISA are highly sensitive but lack specificity, necessitating confirmatory functional assays such as the SRA, which demonstrates >95% specificity [14,15]. In this case, the high optical density and confirmatory SRA established the diagnosis.

The probability of HIT was assessed using the 4Ts scoring system, which evaluates the degree of thrombocytopenia, timing of platelet count decline, presence of thrombosis, and absence of alternative causes [8]. Scores range from 0 to 8, with higher scores indicating an increased likelihood of HIT. In this patient, a score of 8 indicated high pretest probability and prompted immediate cessation of heparin and confirmatory testing.

In our patient, several potential causes of thrombocytopenia were considered before confirming HIT. Sepsis-related disseminated intravascular coagulation (DIC) was unlikely given the absence of fever, leukocytosis, or laboratory evidence of coagulopathy such as prolonged prothrombin time/international normalized ratio or decreased fibrinogen levels. Thrombotic microangiopathies, including thrombotic thrombocytopenic purpura and hemolytic uremic syndrome, were excluded based on the lack of microangiopathic hemolytic anemia, schistocytes on peripheral smear, or renal impairment. The clinical timing of platelet fall (5-10 days post-heparin initiation), magnitude of thrombocytopenia (greater than 50% decline), and new thrombosis, in conjunction with a strongly positive PF4 ELISA and confirmatory SRA, supported the diagnosis of type 2 HIT as the most plausible etiology.

Therapeutic anticoagulation in HIT is essential to prevent thrombotic progression. However, in patients with concurrent bleeding, particularly intracranial hemorrhage, management becomes challenging. In our patient, a non-heparin anticoagulant was initiated, balancing the immediate life-threatening thrombotic risk against the potential for hemorrhagic expansion. Argatroban, a direct thrombin inhibitor, was selected in this case for its rapid onset and short half-life (~45 minutes), allowing for rapid titration and cessation if bleeding occurred or a procedure was warranted. Bivalirudin is another agent with a short half-life [9]. Transition to apixaban, a DOAC, was done once hematoma stability was confirmed. The absence of a loading dose minimized bleeding risk. Recent studies suggest increasing use of DOACs for HIT management, particularly in clinically stable patients once bleeding stability is achieved. The choice between rivaroxaban and apixaban appears driven primarily by institutional preference and familiarity, as both agents have demonstrated similar outcomes [9,16,17].

## Conclusions

This case illustrates the delicate balance between managing thrombosis and mitigating bleeding in patients with HIT and concurrent intracranial hemorrhage. In this patient, careful clinical assessment, multidisciplinary collaboration, and the use of short-acting non-heparin anticoagulants allowed safe and effective management. Future studies should explore integrated models that consider hematologic diseases, such as myeloproliferative neoplasms, and concurrent cardiovascular conditions, such as atrial fibrillation, which amplify both thrombotic and hemorrhagic risk.
